# Combining Whole-Brain Radiotherapy with Gefitinib/Erlotinib for Brain Metastases from Non-Small-Cell Lung Cancer: A Meta-Analysis

**DOI:** 10.1155/2016/5807346

**Published:** 2016-02-24

**Authors:** Mao-hua Zheng, Hong-tao Sun, Ji-guang Xu, Gang Yang, Lei-ming Huo, Pan Zhang, Jin-hui Tian, Ke-hu Yang

**Affiliations:** ^1^Department of Neurosurgery, First Clinical Medical College of Lanzhou University, Lanzhou 730000, China; ^2^Evidence-Based Medicine Center of Lanzhou University, Lanzhou 730000, China; ^3^Key Laboratory of Evidence-Based Medicine and Knowledge Translation of Gansu Province, Lanzhou 730000, China; ^4^Department of Neurosurgery, Affiliated Hospital, Medical College of Chinese People's Armed Police, Tianjin 300162, China

## Abstract

*Background*. To comprehensively assess the efficacy and safety of whole-brain radiotherapy (WBRT) combined with gefitinib/erlotinib for treatment of brain metastases (BM) from non-small-cell lung cancer (NSCLC).* Methods*. Databases including PubMed, EMBASE.com, Web of Science, and Cochrane Library were searched from inception to April 12, 2015. Studies on randomized controlled trials (RCTs) and case-control trials comparing WBRT combined with gefitinib/erlotinib versus WBRT alone for BM from NSCLC were included. Literature selection, data extraction, and quality assessment were performed independently by two trained reviewers. RevMan 5.3 software was used to analyze data.* Results*. A total of 7 trials involving 622 patients were included. Compared with WBRT alone or WBRT plus chemotherapy, WBRT plus gefitinib/erlotinib could significantly improve response rate (OR = 2.16, 95% CI: 1.35–3.47; *P* = 0.001), remission rate of central nervous system (OR = 6.06, 95% CI: 2.57–14.29; *P* < 0.0001), disease control rate (OR = 3.34, 95% CI: 1.84–6.07; *P* < 0.0001), overall survival (HR = 0.72, 95% CI: 0.58–0.89; *P* = 0.002), and 1-year survival rate (OR = 2.43, 95% CI: 1.51–3.91; *P* = 0.0002). In adverse events (III-IV), statistically significant differences were not found, except for rash (OR = 7.96, 95% CI: 2.02–31.34; *P* = 0.003) and myelosuppression (OR = 0.19, 95% CI: 0.07–0.51; *P* = 0.0010).* Conclusions*. WBRT plus gefitinib/erlotinib was superior to WBRT alone and well tolerated in patients with BM from NSCLC.

## 1. Introduction

Brain metastases (BM) are the most frequent intracranial brain tumors, which can be found in approximately 20–40% of all cancer patients. Lung and breast cancers and melanoma are responsible for up to 80% of metastatic brain lesions [1]. Among patients with non-small-cell lung cancer (NSCLC), the proportion that develops brain metastases is as high as 50% [2]. Life expectancy for these patients is poor, and the average survival is 1-2 months without any treatment [3]. A median survival of 3–6 months can be obtained for patients receiving symptomatic therapy with corticosteroids and whole-brain radiotherapy (WBRT) [4].

Recently, several phase II or phase III trials of standard platinum-based chemotherapy regimens for BM from NSCLC have been conducted [5–10], resulting in 23%–68% response rate and 4–12.6-month overall survival. However the effect of combining WBRT with chemotherapy in the management of BM is limited and inconsistent due to the limited ability of most chemotherapeutic drugs to cross the blood brain barrier [11].

In recent years, new targeted therapies are undergoing active development and encouraging results have been obtained so far [12]. A previously published review demonstrated the intracranial efficacy of targeted therapies (EGFR tyrosine kinase inhibitors, ALK inhibitors), which were globally superior to the efficacy of standard chemotherapy [13]. However, this review was qualitative and the quality of evidence was not evaluated. Some retrospective series and phase II randomized studies have been conducted recently to compare the efficacy of combining WBRT with gefitinib/erlotinib versus WBRT alone for patients with BM from NSCLC.

Our study aims therefore to comprehensively assess the quality of currently available evidences and to quantitatively evaluate the efficacy and safety of WBRT combined with gefitinib/erlotinib for BM from NSCLC.

## 2. Methods

This study adhered to the Preferred Reporting Items for Systematic Review and Meta-Analysis (PRISMA) statement [14]. Ethical approval and patient consent were unnecessary for the current study as this was a meta-analysis based on published studies. Literature retrieval, literature selection, data extraction, and quality assessment were performed independently by two trained reviewers; disagreements between the reviewers were resolved by consulting a third expert adjudicator.

### 2.1. Literature Retrieval

Databases retrieved included PubMed, EMBASE.com, Web of Science (via ISI Web of Knowledge), and the Cochrane Library from inception to April 12, 2015, using the terms epidermal growth factor receptor, EGFR, erlotinib, tarceva, erbtinib, iressa, gefitinib, geftinat, brain neoplasm^*∗*^, brain cancer^*∗*^, brain carcinoma^*∗*^, brain tumor^*∗*^, metasta^*∗*^, lung neoplasm^*∗*^, lung cancer^*∗*^, lung carcinoma^*∗*^, lung tumor^*∗*^, whole-brain radiotherapy, WBRT, and other. The references of included studies were tracked to identify potential relevant studies. The search strategy for PubMed was as follows: (((((“lung neoplasm^*∗*^”[Title/Abstract] OR “lung cancer^*∗*^”[Title/Abstract] OR “lung carcinoma^*∗*^”[Title/Abstract] OR “lung tumor^*∗*^”[Title/Abstract] OR “Lung Neoplasms”[Mesh]))) AND ((metasta^*∗*^[Title/Abstract]) AND ((((“brain neoplasm^*∗*^”[Title/Abstract] OR “brain cancer^*∗*^”[Title/Abstract] OR “brain carcinoma^*∗*^”[Title/Abstract] OR “brain tumor^*∗*^”[Title/Abstract]))) OR “Brain Neoplasms”[Mesh]))) AND ((“whole-brain radiotherapy”[Title/Abstract] OR WBRT[Title/Abstract] OR “radiation therapy”[Title/Abstract] OR radiotherapy[Title/Abstract] OR “irradiation therapy”[Title/Abstract] OR “radiation therapy”[Title/Abstract] OR “Radiotherapy”[Mesh]))) AND ((((“epidermal growth factor receptor”[Title/Abstract] OR EGFR[Title/Abstract] OR erlotinib[Title/Abstract] OR tarceva[Title/Abstract] OR erbtinib[Title/Abstract] OR iressa[Title/Abstract] OR gefitinib[Title/Abstract] OR geftinat[Title/Abstract]))) OR “Receptor, Epidermal Growth Factor”[Mesh]).

### 2.2. Inclusion Criteria

Studies meeting the following eligibility criteria were included: (a) type of population: histologically or cytologically confirmed NSCLC and multiple BM (≥3) documented by MRI or contrast CT scan; aged 18 years of age or older; (b) type of intervention: WBRT plus erlotinib/gefitinib; (c) type of comparison: WBRT alone or WBRT plus chemotherapy; (d) type of design: randomized controlled trials (RCTs) or case-control studies; (e) type of outcomes: response rate (RR) and overall survival (OS) were primary endpoints; toxicity, disease control rate (DCR), 1-year survival rate, and remission rate of central nervous system (RR-CNS) were secondary endpoints.

### 2.3. Literature Selection

All records were downloaded and imported into EndNote X6, which is a reference management software tool. Duplicates were removed and the title and abstract of the remaining records were examined independently by two reviewers according to inclusion and exclusion criteria. Then the full texts of potentially relevant studies were obtained to identify interesting studies. Reasons for exclusion were documented.

### 2.4. Data Extraction and Assessment of Risk of Bias

Data were extracted using a predesigned data extraction sheet including the first author, year of publications, sample, median age, intervention regimen, control regimen, study design, outcomes, median OS, and median PFS. Kaplan-Meier curve was read by Engauge Digitizer version 4.1 (available at http://sourceforge.net/) if the adequate data were not reported in the papers [15], and the formula recommended by Spruance et al. [16] was used to calculate the corresponding HR of the missing survival data.

The risk of bias was assessed according to the Cochrane Handbook version 5.1.0 [17], including method of random sequence generation (selection bias), allocation concealment (selection bias), blinding (performance bias and detection bias), incomplete outcome data (detection bias), and selective reporting (detection bias). We evaluated methodological quality as low, high, or unclear risk of bias.

### 2.5. Data Analysis

The odds ratio (OR) with 95% confidence interval (95% CI) was calculated regarding to RR, CN-RR, DCR, 1-year survival rate, and AEs. The Chi-square statistic was used to assess the heterogeneity between trials with *I* ^2^ less than 50% and *P* value greater than 0.10 suggesting that there was no statistical heterogeneity, and a Mantel-Haenszel fixed effects model was used for meta-analysis. A Mantel-Haenszel random effects model was used when clinical characteristics and methodology were not identified to have great difference and *I* ^2^ was greater than 50% and *P* value was less than 0.10. If the clinical characteristic and/or methodology across studies were considered to be obviously different, only qualitative analysis was adopted [18]. Inverse variance fixed or random effects model was used to pool the overall hazard ratio (HR) for OS. When heterogeneity was identified, subgroup analysis and metaregression were conducted to determine the possible causes of heterogeneity such as different target agents (erlotinib or gefitinib), different study designs (randomized or nonrandomized), and sample size (<100 or ≥100). Sensitivity analysis was performed to identify influence of the study regarding overall effective size. In addition, potential publication bias was assessed by using the Begg and Egger tests [19, 20]. *P* value less than 0.05 was considered significant. All data analysis was performed by using RevMan 5.3 software (The Nordic Cochrane Centre, Copenhagen, Denmark) and STATA 12.0 software (Stata Corporation, College Station, Texas, USA).

## 3. Results

### 3.1. Literature Selection and Characteristics of Included Studies

**Figure 1:**
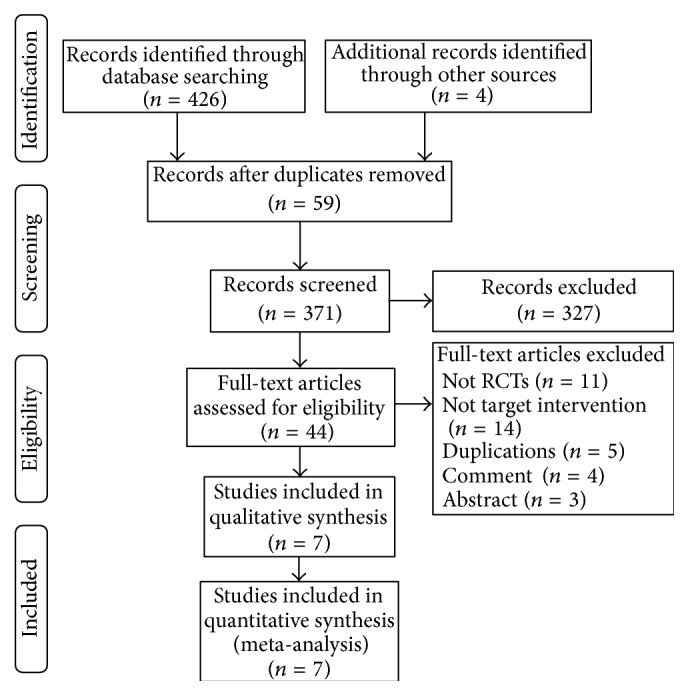
Literature selection flow graph.

A total of 426 records were identified from electronic databases and 4 references were tracked. Finally, 7 studies [21–27] involving 622 patients were included. The search results and selection details are shown in Figure 1.

The detailed characteristics of included studies are shown in Table 1. Of the seven studies included, three [22, 24, 26] were randomized controlled trials and four [21, 23, 25, 27] were case-control studies. They were published between 2012 and 2014. The sample sizes ranged from 53 to 161. Three studies could be identified as phase II and four did not report trial phase. Median OS was reported in five studies [22, 24–27], and only two [22, 27] reported median nPFS.

### 3.2. Assessment of Risk of Bias

**Figure 2:**
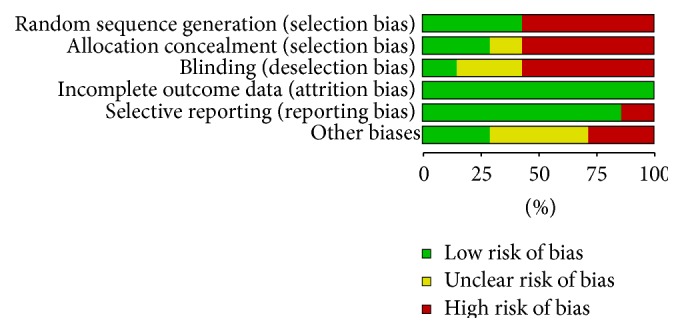
Analysis of risk of bias.

The details of this analysis are shown in Figure 2. Four studies were case-control trials; therefore the risk of bias was high regarding adequate sequence generation, adequate allocation concealment, and blinding. Of three RCTs included, all described methods for adequate sequence generation such as center random and minimization method; two RCTs reported using adequate allocation concealment, and one RCT was a double-blinded design study. The overall methodological quality of included studies was poor because only three were RCTs.

## 4. Results of Meta-Analysis

### 4.1. Response Rate (RR)

**Figure 3:**
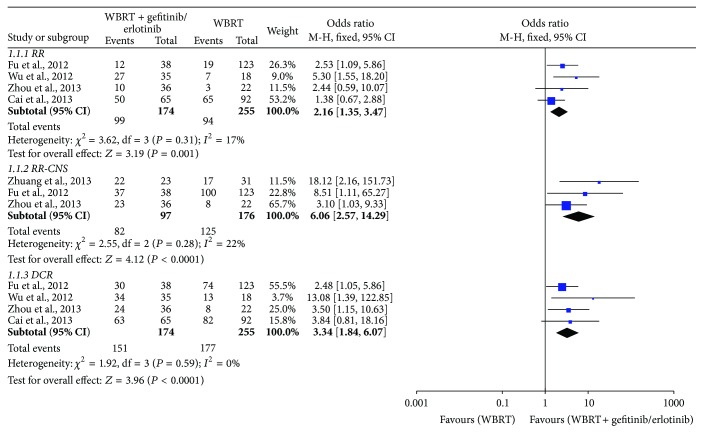
Meta-analysis for RR, RR-CNS, and DCR.

Four studies [23–25, 27] reported the overall response rate for patients with BM. The heterogeneity test indicated that a fixed effects model could be used to pool the RR (*I* ^2^ = 17%, *P* = 0.31). Compared with WBRT alone, there was a statistically significant improvement in RR for WBRT combined with gefitinib/erlotinib (OR = 2.16, 95% CI: 1.35–3.47; *P* = 0.001) (Figure 3).

Three studies [21, 23, 25] reported the RR-CNS, with 273 patients involved. There was no significant statistical heterogeneity in pooled analysis of all included studies (*I* ^2^ = 22%, *P* = 0.28) and thus a fixed effects model was used to perform meta-analysis. Compared with WBRT alone, there was a statistically significant improvement in RR-CNS for WBRT combined with gefitinib/erlotinib (OR = 6.06, 95% CI: 2.57–14.29; *P* < 0.0001) (Figure 3).

### 4.2. Disease Control Rate (DCR)

Four studies [23–25, 27] reported the overall response rate, with 429 patients involved. The heterogeneity test indicated that a fixed effects model could be used (*I* ^2^ = 0%, *P* = 0.59). Compared with WBRT alone, there was a statistically significant improvement in DCR for WBRT combined with gefitinib/erlotinib (OR = 3.34, 95% CI: 1.84–6.07; *P* < 0.0001) (Figure 3).

### 4.3. Overall Survival (OS)

**Figure 4:**
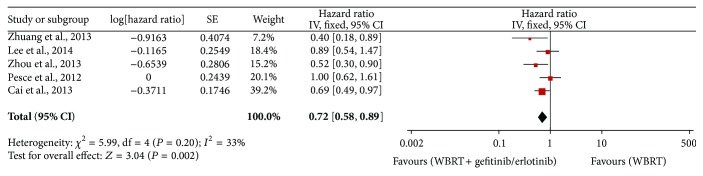
Meta-analysis for OS.

Five studies [21, 22, 25–27] reported the overall survival, with 408 patients involved. The heterogeneity test indicated that a fixed effects model could be used (*I* ^2^ = 33%, *P* = 0.20). The meta-analysis showed that WBRT combined with gefitinib/erlotinib significantly prolonged OS compared to WBRT alone (HR = 0.72, 95% CI: 0.58–0.89; *P* = 0.002) (Figure 4).

### 4.4. 1-Year Survival Rate

**Figure 5:**
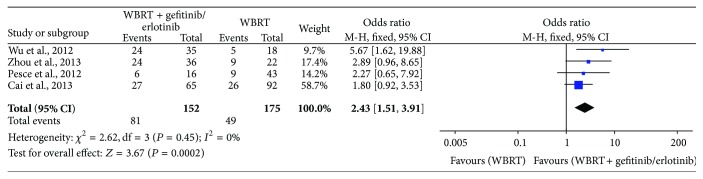
Meta-analysis for 1-year survival rate.

Four studies [24–27] reported the 1-year survival rate, with 327 patients involved. The heterogeneity test indicated that a fixed effects model could be used (*I* ^2^ = 0%, *P* = 0.45). The meta-analysis showed that WBRT combined with gefitinib/erlotinib significantly prolonged 1-year survival rate compared to WBRT alone (OR = 2.43, 95% CI: 1.51–3.91; *P* = 0.0002) (Figure 5).

### 4.5. Adverse Events (III-IV)

**Figure 6:**
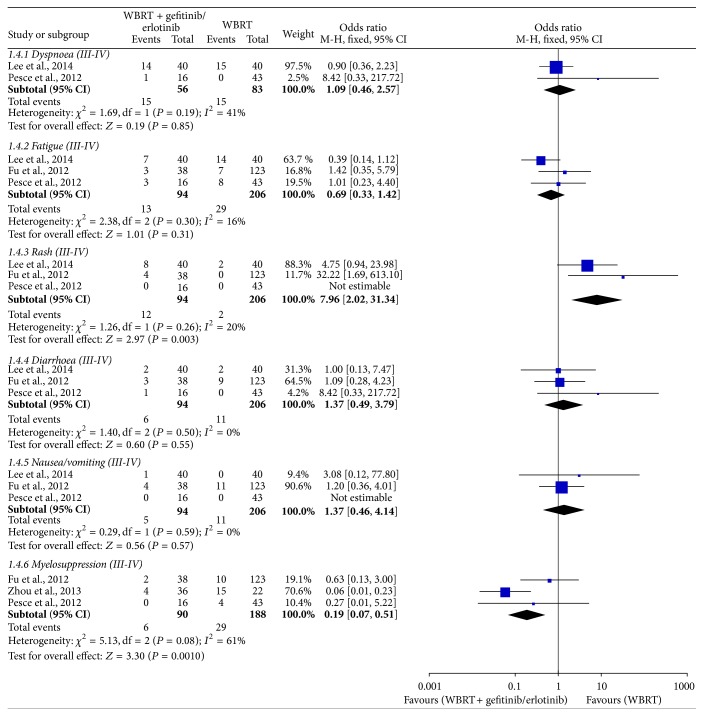
Meta-analysis for adverse events (III-IV).

The results of the meta-analysis for adverse events are shown in Figure 6. The heterogeneity tests for all adverse events indicated that there were no statistical differences except for myelosuppression (III-IV) (*I* ^2^ < 50%, *P* > 0.10). The meta-analysis showed that WBRT plus gefitinib/erlotinib increased the incidence of rash (III-IV) (OR = 7.96, 95% CI: 2.02–31.34; *P* = 0.003) but reduced the incidence of myelosuppression (III-IV) (OR = 0.19, 95% CI: 0.07–0.51; *P* = 0.0010). Statistical differences were not found regarding other adverse events.

### 4.6. Subgroup Analysis and Sensitivity Analysis

The heterogeneity tests for interesting outcomes indicated that there were no statistical differences between studies (*I* ^2^ < 50%, *P* > 0.10). Therefore subgroup analysis and meta regression were not conducted for the current study.

**Figure 7:**
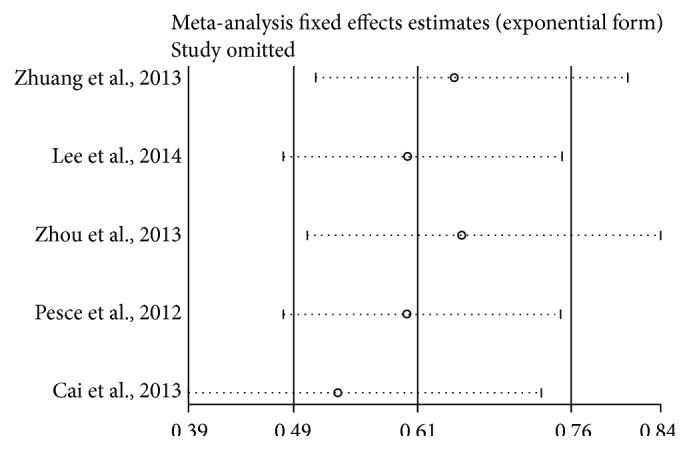
Sensitivity analysis of OS.

Figure 7 shows the results of sensitivity analysis regarding OS. We found that excluded studies did not influence the overall effective size.

### 4.7. Publication Bias

For the meta-analyses of RR and OS, there was no evidence of significant publication bias by inspection of the formal statistical tests (RR: Egger's test, *P* = 0.276; Begg's test, *P* = 0.497 and OS: Egger's test, *P* = 0.478; Begg's test, *P* = 0.142).

## 5. Discussion

This is a systematic review and meta-analysis to comprehensively assess the efficacy and safety of WBRT combined with gefitinib/erlotinib for treatment of BM from NSCLC. The present meta-analysis suggests that compared with WBRT alone or WBRT plus chemotherapy, WBRT plus gefitinib/erlotinib can significantly improve the RR, RR-CNS, and DCR and prolong the OS and 1-year survival rate. Regarding the incidences of adverse events, WBRT plus gefitinib/erlotinib was well tolerated except for increased risk of rash (III-IV) in the treatment of patients with multiple BM from NSCLC.

Current therapeutic approaches for patients with multiple BM from NSCLC mainly include surgery, whole-brain radiotherapy (WBRT), stereotactic radiosurgery (SRS), and chemotherapy [28]. However, advances in the understanding of the BM pathobiology and development of molecular targeted agents hold promise for improved prophylaxis and therapy of BM [29]. Epidermal growth factor receptor (EGFR) tyrosine kinase inhibitors (TKIs), such as erlotinib, have been approved in 2004 by the US Food and Drug Administration for treating locally advanced or metastatic NSCLC [30]. A mass of studies have demonstrated that the objective response rate is 42.9%–87.5%, and DCR is as high as 87.5%–100.0% for targeted agents combined with radiotherapy in the treatment of patients with multiple BM [31]. A phase II trial also showed that the median overall survival time was 11.8 months for patients with BM from NSCLC [32]. These results are consistent with Zhuang et al. [21]. However, Pesce et al. [26] concluded that WBRT plus gefitinib could not prolong the survival time for patients with BM from NSCLC. The discrepancy can be ascribed to the limited sample size of Pesce et al.'s study. A meta-analysis was therefore urgently needed to systematically assess the quality of available evidence and make a scientific conclusion about WBRT plus gefitinib/erlotinib in treating BM from NSCLC.

Regrettably, a subgroup meta-analysis related to EGFR mutation status was not conducted because only one trial was identified. Previous studies showed that targeted therapy was beneficial for patients with BM. The overall response rate was 70%–89% for patients with intracranial lesions, and the overall survival time was 12.9–19.8 months longer [33, 34]. Zhuang et al.'s study showed that, compared with EGFR wild-type patients, there was no significant improvement in LPFS, PFS, and OS for mutated EGFR mutation patients [21]. More studies of WBRT plus gefitinib/erlotinib in treatment of BM with EGFR mutations are needed.

The present study had certain limitations. Firstly, the overall methodological quality of included studies was low. Only three RCTs were included to assess the efficacy of the combined treatment. Most of the studies evaluating WBRT plus gefitinib/erlotinib for BM from NSCLC were case series, and only few controlled trials could be identified. Secondly, the small sample size of the included studies might have led to inadequate statistical power. The present conclusions are based on phase II trials, and more phase III randomized controlled trials are needed. Thirdly, subgroup analyses of different pathological subtypes, trial phase, smoking status, median age, and EGFR mutations status were not performed due to inadequate reporting across studies.

Overall, the currently available evidence indicates that RR, RR-CNS, DCR, OS, and 1-year survival rate can be improved by using WBRT combined with gefitinib/erlotinib in patients with BM from NSCLC, and the adverse events (III-IV) are well tolerated. Moreover, the efficacy of other targeted agents for BM from NSCLC should be assessed in future studies.

## Figures and Tables

**Table 1:** Characteristics of included studies.

Studies	Samples		Median age (years)		Intervention regimens	Control regimens	Design	Phase	Median OS (months)	Median nPFS (months)
	W-T	W	W-T	W						
Zhuang et al., 2013 [21]	23	31	60 (37–76)	63 (43–81)	WBRT 30 Gy/10 f plus erlotinib 150 mg/day	WBRT 30 Gy/10 f	Case-control	II	NR	NR

Lee et al., 2014 [22]	40	40	61.3 (48–75)	62.2 (41–73)	WBRT 20 Gy/5 f plus erlotinib 100 mg/day	WBRT 20 Gy/5 f + placebo	Randomized	II	3.4/2.9	1.6/1.6

Fu et al., 2012 [23]	38	123	A56 (38–77)		WBRT 30–40 Gy/2-3 W plus gefitinib 250 mg/day	WBRT 30–40 Gy/2-3 W	Case-control	NR	NR	NR

Wu et al., 2012 [24]	35	18	18–65		WBRT 40 Gy/20 f plus gefitinib 250 mg/day	WBRT 40 Gy/20 f	Randomized	NR	12.1/9.8	NR

Zhou et al., 2013 [25]	36	22	27–75		WBRT 40 Gy/20 f or 30 Gy/10 f plus gefitinib 250 mg/day or erlotinib 100 mg/day	WBRT 40 Gy/20 f or 30 Gy/10 f plus Taxol 135–175 mg/m^2^ d1 or Alimta 500 mg/m^2^ d1 or DDP 25 mg/m^2^ (d1–3)	Case-control	NR	23.2/7.1	NR

Pesce et al., 2012 [26]	16	43	57 (46–82)	63 (45–79)	WBRT 30 Gy/10 f plus gefitinib 250 mg/day	WBRT 30 Gy/10 f plus TMZ 75 mg/m^2^/day	Randomized	II	6.3/4.9	NR

Cai et al., 2013 [27]	65	92	66 (35–81)		WBRT 29.37~41.24 Gy, 3 Gy/d, 5 times/week plus gefitinib 250 mg/day or erlotinib 100 mg/day	WBRT 29.37~41.24 Gy, 3 Gy/d, 5 times/week	Case-control	NR	10.6/7.7	6/3.4

*Notes*. W-T: WBRT plus erlotinib/gefitinib; W: WBRT; NR: not reported; OS: overall survival; PFS: progression-free survival.
